# Impact of Pictorial Health Warnings on Cigarette Packs among Current Smokers of Sub-Metropolitan City of Nepal: A Descriptive Cross-sectional Study

**DOI:** 10.31729/jnma.8743

**Published:** 2024-09-30

**Authors:** Ashish Shrestha, Tarakant Bhagat, Santosh Kumari Agrawal, Binita Limbu

**Affiliations:** 1 Department of Public Health Dentistry, College of Dental Surgery, B.P. Koirala Institute of Health Sciences, Ghopa, Dharan, Nepal

**Keywords:** *pictorial health warning*, *quit intention*, *smoking*, *smoking cessation*

## Abstract

**Introduction:**

Pictorial health warning on cigarette packs is one of the strategies undertaken by Nepal Government as per the directive of World Health Organization Framework Convention on Tobacco Control to control smoking. It has been more than a decade since the introduction of pictorial health warnings on cigarette packs in Nepal. The objective of this study was to assess the impact of the pictorial warnings among current smokers of Dharan, Nepal.

**Methods:**

A descriptive cross-sectional study was conducted among current smokers visiting local shops located near ward offices of Dharan after obtaining ethical approval from Institutional Review Committee (Reference number: 204/080/01 -IRC). A structured and validated Nepali questionnaire was used to interview the smokers about the impact of the pictorial warnings based on their noticeability, behavioural and cognitive responses. Statistical analysis was done using Statistical Package for Social Sciences (SPSS) version 20.0. The responses of the participants were tabulated in frequencies and percentages.

**Results:**

The pictorial warnings were noticed by 277 (98.93%) participants in past one month out of which 11 (3.93%) smokers had always read or looked closely at them. Smoking less frequently was the behaviour change reported by 70 (25%) participants and smoking less around others was reported by and 56 (20%) participants. In total, 189 (67.50%) smokers had thought about the harms of smoking and 160 (57.14%) had intention to quit smoking due to the pictorial warnings.

**Conclusions:**

The pictorial health warnings were not so effective in bringing about changes in smoking behaviour of smokers.

## INTRODUCTION

Tobacco is the second most common risk factor for deaths and third most common risk factor for total disability-adjusted life years (DALYs) in Nepal.^1^ Nepal Government has mandated printing of pictorial health warnings covering at least 90% of tobacco product packaging to control tobacco use.^2^ Large graphic warnings on tobacco products provide information about health risks of tobacco and also reduce any attraction associated with the product.^3^

A systematic review on the effect of pictorial health warnings (PHWs) on cigarette packages on smoking behaviour has found conflicting findings.^4^ Previous study of Nepal showed the warnings to be effective in motivating smokers to quit smoking. Further studies regarding the impact of these warnings was suggested.^5^

It has been more than a decade since pictorial warnings have been displayed in cigarette packages. Hence, this study was undertaken to assess the effectiveness of pictorial warnings among current smokers of Dharan city of Nepal.

## METHODS

It was a descriptive cross-sectional study conducted among current smokers of the Dharan sub-metropolitan city of Nepal from 1^st^ November, 2023 to 31^st^ January, 2024. Ethical approval for the study was obtained from the Institutional Review Committee, B.P. Koirala Institute of Health Sciences (BPKIHS), Dharan (Ref. No.204/080/01-IRC). Informed consent was obtained from all the participants. Individuals aged 18 years and above who were current smokers (a person who smokes cigarette on a daily basis) were included in the study whereas those not willing to participate in the study were excluded.

The sample size was calculated using the following formula:


$$\begin{array}{ll} \text{n} & ={\text{Z}}^{2}\times \frac{\text{p}\times \text{q}}{{\text{e}}^{\text{2}}}\\ & =1.96^{2}\times \frac{0.802\times 0.198}{0.05^{2}}\\ & =244 \end{array}$$

Where,

n = minimum required sample size for infinite population
z = 1.96 at 95% Confidence Interval (CI)
p = 80.2%, prevalence among general population^6^
e = margin of error, 5%
q = 100-p

Considering 10% non-response rate, n = 268

The required sample size was 268. For equal allocation, 14 smokers were recruited from all the 20 wards so total of 280 smokers were enrolled in this study. Local shops near each ward office of Dharan were chosen conveniently and equal number of smokers visiting those shops to buy cigarettes were selected for the study.

They were interviewed face-to-face using a structured questionnaire adapted from Global Adult Tobacco Survey (GATS) questionnaire and from studies done by Borland et al. and Mishra et al. the questionnaire was translated and validated in Nepali language using standard WHO guidelines.^6-8^ The questionnaire consisted of 11 questions. First part of the questionnaire addressed the demographic characteristics of the participants. Other questions assessed the smoking behaviour of the participants and the impact of pictorial health warnings based on the noticeability, behavioural and cognitive responses (thought of harm of smoking and intention to quit). Face and content validity of the questionnaire was assessed before and after translating the questionnaire into Nepali language. The translated questionnaire was pre-tested on 30 current smokers who visited dental out patient department of BPKIHS for their dental treatment to check the comprehensibility and reliability of the questionnaire. The value of Cronbach's alpha was 0.74. Statistical analysis was done using Statistical Package for Social Sciences (SPSS) version 20.0. The responses of the participants were tabulated in frequencies and percentages.

## RESULTS

Among 280 current smokers, 258 (92.14%) were male. The age of the study participants ranged from 18 to 76 years with median age of 36 years. There were 176 (62.85%) participants having a high school degree or above, 99 (35.36%) had started daily smoking at the age of 15 to 18 years and 176 (62.86%) smoked less than 10 cigarettes per day. Out of total, 116 (41.43%) participants smoked first cigarette after more than an hour of waking. The number of participants who bought cigarettes in loose form was 193 (68.93%). Among all, 154 (55%) had made attempts to quit smoking in the past 12 months and 241 (86.07%) of them believed that smoking causes serious illness (Table 1).

**Table 1:** Smoking characteristics of the study participants (n=280).

Variables	n (%)
**Age at daily smoking initiation**	
Less than 15 years	45 (16.07)
15-18 years	99 (35.36)
19-21 years	60 (21.43)
22 years and above	76 (27.14)
**Average number of cigarettes smoked per day**	
Less than 5	87 (31.07)
5-9	89 (31.79)
10-14	45 (16.07)
15-20	50 (17.86)
More than 20	9 (3.21)
**Time of first smoking upon waking**	
Within 5 minutes	86 (30.71)
6-30 minutes	43 (15.36)
31-60 minutes	35 (12.50)
>60 minutes	116 (41.43)
**How cigarettes are bought**	
Loose	193 (68.93)
Packets	87 (31.07)
**Smoking quit attempts made in past 12 months**	
Yes	154 (55)
No	126 (45)
**Belief that smoking causes serious illness**	
Yes	241 (86.07)
No	39 (13.93)

Amongst the total participant, 111 (39.64%) had noticed Pictorial Health Warnings (PHWs) on cigarette packs, 94 (33.57%) read or looked at it closely, 189 (67.50%) had thought about the harms of smoking and 160 (57.14%) has thought about the quitting smoking due to PHWs (Table 2).

**Table 2:** Noticeability and impact of pictorial health warnings among the current smokers of Dharan (n = 280).

Variables	n (%)
**Noticed PHWs**	
Never	3 (1.07)
Rarely	8 (2.86)
Sometimes	90 (32.14)
Very often	68 (24.29)
Always	111 (39.64)
**Read or looked closely at PHWs**	
Never	66 (23.57)
Rarely	62 (22.14)
Sometimes	94 (33.57)
Very often	47 (16.79)
Always	11 (3.93)
**Behaviour changes due to PHWs**	
Smoked less around others	56 (20)
Covered the pack	14 (5)
Kept pack out of sight	10 (3.57)
Smoked less frequently	70 (25)
Stubbed out a cigarette	8 (2.86)
No change in behaviour	122 (43.57)
**Thought about harms of smoking due to PHWs**	
Yes	189 (67.50)
No	91 (32.50)
**Thought about quitting smoking due to PHWs**	
Yes	160 (57.14)
No	120 (42.86)
PHWs: Pictorial health warnings	

## DISCUSSION

Our study showed high noticeability of pictorial health warnings on cigarette packs as almost all participants reported to have seen the warnings in past one month. Similar result was shown by previous studies.^5,9-11^ It has been more than a decade of introduction of PHWs in cigarette packs hence, high noticeability of the warnings was expected in our study. Despite the high noticeability of PHWs, less proportion of the smokers reported to have read or looked closely at the warnings. In a study done in western Nepal, 66.3% participants had thought about what PHWs had to say.^11^

Pictorial health warnings were found to have minimal impact on smoking behaviour of the study participants. Only one-fifth of the participants reported to smoke less around others while one-fourth reported to smoke less frequently due to the warnings. Previous studies done in Nepal by Shrestha et al.^11^ and Mishra et al.^8^ had 20.1% and 12% of smokers that smoked less around others due to the warnings. In our study, the proportion of participants who covered the pack or kept it out of sight was less and it was comparable to that of the previous study of Nepal.^8^ Contrary to this, study of Shrestha et al. had 50.9% of smokers that avoided looking at the warning labels.^11^ Avoidance of warnings may appear to be a negative response but it is found to be positively associated with quitting. However, it may not be a consistent predictor of quit attempts.^12^ The reason of failure of PHWs to bring about changes in smoking behaviour could be attributed to less attentiveness of smokers towards the warnings. It could also be due to immediate reinforcing power of smoking. Changing habitual behaviours and turning the intention to quit into actual and sustained behavioural change is very difficult.^4,13^

In our study, 189 (67.50%) of smokers had thought about the harms of smoking due to the PHWs which was lower than previous study of Nepal (85.8%).^11^ Studies have shown that the effects of the warnings may wear off over time.^7,14^ Likewise, 160 (57.14%) of our participants had the thought of quitting smoking due to the pictorial health warnings. Shrestha et al.^11^ reported that 40.2% of smokers perceived PHWs to be effective in motivating smokers to quit. Studies have reported similar proportion of smokers with intention for smoking cessation due to PHWs.^5,10,15^ PHWs increase quit intentions by increasing attention to physical harm warnings, prompting emotional response, evoking negative reactions, avoiding warnings and taking the warning message into consideration.^15^ They present the harming potential of smoking in more realistic manner and make smokers think about acting to avoid the risk.^16^

The present study had several limitations. The sample was selected conveniently among the smokers who visited local shops to buy cigarettes so it may not be representative of all the current smokers of Dharan limiting the generalizability of the study findings. The information regarding smoking characteristics of the smokers and impact of PHWs was self-reported and based on memory. So, there is possibility of recall bias or response bias. Furthermore, the impact of PHWs was assessed in terms of self-reported behaviour change and intention to quit. The real effect of PHWs on smoking behaviour or quitting attempts could not be assessed in this study.

## CONCLUSIONS

The PHWs were not so effective in bringing about changes in smoking behaviour of smokers. However, the warnings were impactful in terms of cognitive processing among the smokers.

## References

[1] Shrestha G, Phuyal P, Gautam R, Mulmi R, Pradhan PMS (2021). Burden of tobacco in Nepal: a systematic analysis from the Global Burden of Disease Study 1990-2017.. BMJ Open..

[2] Government of Nepal. Directive for Printing and Labeling of Warning Message and Picture on the Box, Packet, Wrapper, Carton, Parcel and Packaging of Tobacco Product -2014..

[3] World Health Organization. (2013). Pictorial Warnings Article 11: Packaging and labelling of tobacco products..

[4] Monärrez-Espino J, Liu B, Greiner F, Bremberg S, Galanti R (2014). Systematic Review of the Effect of Pictorial Warnings on Cigarette Packages in Smoking Behavior.. American Journal of Public Health..

[5] Bam TS, Chand AB, Shah BV (2021). Evidence of the Effectiveness of Pictorial Health Warnings on Cigarette Packaging in Nepal.. Asian Pacific Journal of Cancer Prevention..

[6] GATS Questionnaire. https://www.who.int/teams/noncommunicable-diseases/surveillance/systems-tools/global-adult-tobacco-survey/questionnaire.

[7] Borland R, Wilson N, Fong GT, Hammond D, Cummings KM, Yong HH (2009). Impact of graphic and text warnings on cigarette packs: findings from four countries over five years.. Tobacco Control..

[8] Mishra SR, Bhandari PM, Marahatta SB, Rana HKS, Acharya P, Khanal V (2016). Current smokers' perception of cigarette graphic health warnings and smoking habits: a cross sectional study from Nepal.. Health Prospect: Journal of Public Health..

[9] Tripathy JP, Verma M (2022). Impact of Health Warning Labels on Cigarette Packs in India: Findings from the Global Adult Tobacco Survey 2016-17.. Behavioral Medicine..

[10] Ngoc Bich N, Thu Ngan T, Bao Giang K, Thi Hai P, Thi Thu Huyen D, Ngoc Khue L (2019). Salience and impact of health warning label on cigarette packs in Vietnam: Findings from the Global Adult Tobacco Survey 2015.. Behavioural Medicine..

[11] Shrestha S, Pokhrel S, Subedi S, Paudel H, Viswakarma RC, Poudel D (2022). Perception of cigarette graphic health warnings and its impact on smoking behaviour: A cross-sectional study among current smokers of western part of Nepal.. Journal of Smoking Cessation..

[12] Borland R, Yong H, Wilson N, Fong GT, Hammond D, Cummings KM (2009). How reactions to cigarette packet health warnings influence quitting: findings from the ITC Four-Country survey.. Addiction..

[13] Malouff JM, Schutte NS, Rooke SE, MacDonell G (2012). Effects on smokers of exposure to graphic warning images.. The American Journal on Addictions..

[14] Li L, Borland R, Yong H, Cummings KM, Thrasher JF, Hitchman SC (2015). Longer term impact of cigarette package warnings in Australia compared with the United Kingdom and Canada.. Health Education Research..

[15] Wang R, Qiang Y, Zhu Y, Gao X, Yang Q, Li B (2021). The estimated effect of graphic warning labels on smoker's intention to quit in Shanghai, China: a cross-sectional study.. BMC Public Health..

[16] Fathelrahman AI, Omar M, Awang R, Cummings KM, Borland R, Samin ASBM (2010). Impact of the new malaysian cigarette pack warnings on smokers' awareness of health risks and interest in quitting smoking.. International Journal of Environmental Research and Public Health..

